# Supplementary material for: Initial dose reduction of enzalutamide does not decrease the incidence of adverse events in castration-resistant prostate cancer
Source: PLoS One. 2021 Oct 1;16(10):e0258160. doi: 10.1371/journal.pone.0258160 (PMC8486121; doi:10.1371/journal.pone.0258160)
Supplement: Attachment — Submitted filename: Response to Reviewer.doc [file pone.0258160.s002.doc]

Manuscript #: PONE-D-21-17359

Title: Initial dose reduction of enzalutamide does not decrease the incidence of adverse events in castration-resistant prostate cancer

Dear Editor,

Thank you for reviewing our manuscript and offering us the opportunity to submit the revised one. We would also like to thank PLOS ONE Editorial Board that provides an opportunity to improve the manuscript with their helpful comments. We have revised the manuscript according to the reviewers’ suggestions. Below are our responses to the reviewers’ comments.

Sincerely yours,

Shunsuke Tsuzuki

Takahiro Kimura

(On-behalf of all co-authors)

Associate Editor's comments:

1) Please ensure that your manuscript meets PLOS ONE's style requirements, including those for file naming. The PLOS ONE style templates can be found at 
<https://journals.plos.org/plosone/s/file?id=wjVg/PLOSOne_formatting_sample_main_body.pdf> and 
<https://journals.plos.org/plosone/s/file?id=ba62/PLOSOne_formatting_sample_title_authors_affiliations.pdf>

REPLY: We thank the associate editor for his/her comment. We revised the manuscript as following the PLOS ONE’s style requirements including those for file naming.

2) Thank you for stating the following in the Competing Interests section:

I have read the journal's policy and the authors of this manuscript have the following competing interests:Shin Egawa is a paid consultant/advisor of Takeda, Astellas, AstraZeneca, Sanofi, Janssen, and Pfizer. Takahiro Kimura is a paid consultant/advisor of Astellas, Bayer, Janssen and Sanofi.

Please confirm that this does not alter your adherence to all PLOS ONE policies on sharing data and materials, by including the following statement: "This does not alter our adherence to  PLOS ONE policies on sharing data and materials.” (as detailed online in our guide for authors <http://journals.plos.org/plosone/s/competing-interests>).  If there are restrictions on sharing of data and/or materials, please state these. Please note that we cannot proceed with consideration of your article until this information has been declared.

Please include your updated Competing Interests statement in your cover letter; we will change the online submission form on your behalf.

REPLY: We thank the associate editor for his/her comment. We moved the statement of competing interests into our revised covering letter and added the following sentence: This does not alter our adherence to PLOS ONE policies on sharing data and materials.

3) We note that you have indicated that data from this study are available upon request. PLOS only allows data to be available upon request if there are legal or ethical restrictions on sharing data publicly. For more information on unacceptable data access restrictions, please see [http://journals.plos.org/plosone/s/data-availability#loc-unacceptable-data-access-restrictions](http://journals.plos.org/plosone/s/data-availability#loc-unacceptable-data-access-restrictions).

In your revised cover letter, please address the following prompts:

a) If there are ethical or legal restrictions on sharing a de-identified data set, please explain them in detail (e.g., data contain potentially sensitive information, data are owned by a third-party organization, etc.) and who has imposed them (e.g., an ethics committee). Please also provide contact information for a data access committee, ethics committee, or other institutional body to which data requests may be sent.

b) If there are no restrictions, please upload the minimal anonymized data set necessary to replicate your study findings as either Supporting Information files or to a stable, public repository and provide us with the relevant URLs, DOIs, or accession numbers. For a list of acceptable repositories, please see [http://journals.plos.org/plosone/s/data-availability#loc-recommended-repositories](http://journals.plos.org/plosone/s/data-availability#loc-recommended-repositories).

We will update your Data Availability statement on your behalf to reflect the information you provide.

REPLY: We thank the associate editor for his/her comment. Since there is no restriction to share the anonymized data set, we added the following sentence to the revised covering letter: We uploaded the anonymized data set as a Supporting file. Then, we uploaded the data set as a Supporting file.

4)  We note you have included a table to which you do not refer in the text of your manuscript. Please ensure that you refer to Table 6 in your text; if accepted, production will need this reference to link the reader to the Table.

REPLY: We thank the associate editor for his/her comment. We put every Tables 1-6 in the revised manuscript as the PLOS ONE’s style requirements Then we highlighted the part we referred the Table6 in the revised manuscript.

5) Please review your reference list to ensure that it is complete and correct. If you have cited papers that have been retracted, please include the rationale for doing so in the manuscript text, or remove these references and replace them with relevant current references. Any changes to the reference list should be mentioned in the rebuttal letter that accompanies your revised manuscript. If you need to cite a retracted article, indicate the article’s retracted status in the References list and also include a citation and full reference for the retraction notice.

REPLY: We thank the associate editor for his/her comment. We reviewed our reference list to ensure that it is complete and correct.

Reviewer #1: The authors have explored the impact of the initial dose of enzalutamide on the treatment response and incidence of AEs in patients with CRPC. They report that initial dose modification of enzalutamide did not decrease the incidence of AEs and might impair the efficacy. The topic is clinically significant as the patients are getting older, as written in the manuscript. The manuscript is concise and well-written. Some points to be clarified are as below.

1) In the univariate and multivariate analysis, 'Time to CRPC <12m' is independent risk factor for the prediction of PSA progression-free survival (Table 4). Then, I suppose some patients who had long, durable response with previous ADT (Time to CRPC >12m) might be treated with initial dose reduction. In contrast, some with resistant to ADT might be treated without dose modification. So , Could you check and provide subgroup analysis between these two group according to Time to CRPC?

REPLY: We thank the reviewer for his/her important indication. However, as presented in Table 1, the time to CRPC was not significantly different between the groups and the median tended to be shorter in the reduced dose group than the standard dose group, actually (16.5 vs 19.5 months, respectively). According to the reviewer’s suggestion, the subgroup analyses were conducted. The patients were divided into groups with time to CRPC ≥12 months (n=74) and <12months (n=27) and PSA response was compared between the reduced and standard dose groups. As shown below, in the group with time to CRPC ≥12 months, PSA response was better in the standard dose group than in the reduced dose group which was consistent to the results of the entire cohort (Table 1). However, in the group with time to CRPC < 12months, PSA response was not different regardless of initial dose (Table 2). We assumed it was mainly caused by the limited number of patients in this subgroup.

Table 1 Time to CRPC ≥12 months

Table 2 Time to CRPC <12months

We added the following description: Although it was not statistically significant difference, the median time to CRPC in reduced dose group was shorter than that of the standard dose group (16.5 vs 19.5months, respectively). in the result section.

2) It would be good to provide relative dose intensity if you can calculate from the subjects.

REPLY: We really appreciate the reviewer for his/her important indication. However, we are sorry to say that we cannot calculate RDI because detailed data about prescription of enzalutamide in individual patients are not available, unfortunately.

3) In Table 2, the Median time to CRPC (IQR) of the two groups seems different (median time 10.5 vs. 20.5). Although, P-value is not statistically significant. I recommend checking this value and provide other values, such as mean time or distribution if possible.

REPLY: We thank the reviewer for his/her comment. In the Table2, we did the wrong typing about the median time to CRPC in the reduced dose group. The correct number was 15.0 month. Moreover, mean and distribution of time to CRPC in the standard dose and the reduced dose groups were 29.6 vs 30.8 months and 3-151 vs 1-127 months, respectively (p=0.58).

Change)

We changed the number to 15.0 in Table2.

4) Please add abbreviations to each table.

REPLY: We thank the reviewer for his/her comment. We added the abbreviations to each Table1-6.
